# Recent advances in Tumor Treating Fields (TTFields) therapy for glioblastoma

**DOI:** 10.1093/oncolo/oyae227

**Published:** 2024-10-14

**Authors:** Simon Khagi, Rupesh Kotecha, Na Tosha N Gatson, Suriya Jeyapalan, Huda Ismail Abdullah, Nicholas G Avgeropoulos, Eleni T Batzianouli, Moshe Giladi, Leonardo Lustgarten, Samuel A Goldlust

**Affiliations:** Hoag Family Cancer Institute, Newport Beach, CA, United States; Department of Radiation Oncology, Miami Cancer Institute, Baptist Health South Florida, Miami, FL, United States; Neuro-Oncology Center of Excellence, Indiana University School of Medicine, Indianapolis, IN, United States; IU Health Neuroscience & Simon Cancer Institutes, Indianapolis, IN, United States; Geisinger Commonwealth School of Medicine, Scranton, PA, United States; Tufts Medical Center, Boston, MA, United States; Novocure Inc. New York City, NY, United States; Novocure Inc. New York City, NY, United States; Novocure GmBH, Root, Switzerland; Novocure Ltd, Haifa, Israel; Novocure Inc. New York City, NY, United States; Department of Neuro-Oncology, Saint Luke’s Cancer Institute, Kansas City, MO, United States

**Keywords:** Tumor Treating Fields, glioblastoma, brain tumor, alternating electric field, antitumor immunity, medical device

## Abstract

Tumor Treating Fields (TTFields) therapy is a locoregional, anticancer treatment consisting of a noninvasive, portable device that delivers alternating electric fields to tumors through arrays placed on the skin. Based on efficacy and safety data from global pivotal (randomized phase III) clinical studies, TTFields therapy (Optune Gio) is US Food and Drug Administration-approved for newly diagnosed (nd) and recurrent glioblastoma (GBM) and Conformité Européenne-marked for grade 4 glioma. Here we review data on the multimodal TTFields mechanism of action that includes disruption of cancer cell mitosis, inhibition of DNA replication and damage response, interference with cell motility, and enhancement of systemic antitumor immunity (adaptive immunity). We describe new data showing that TTFields therapy has efficacy in a broad range of patients, with a tolerable safety profile extending to high-risk subpopulations. New analyses of clinical study data also confirmed that overall and progression-free survival positively correlated with increased usage of the device and dose of TTFields at the tumor site. Additionally, pilot/early phase clinical studies evaluating TTFields therapy in ndGBM concomitant with immunotherapy as well as radiotherapy have shown promise, and new pivotal studies will explore TTFields therapy in these settings. Finally, we review recent and ongoing studies in patients in pediatric care, other central nervous system tumors and brain metastases, as well as other advanced-stage solid tumors (ie, lung, ovarian, pancreatic, gastric, and hepatic cancers), that highlight the broad potential of TTFields therapy as an adjuvant treatment in oncology.

## Teaser

This article reviews the mechanism, efficacy, safety, and quality of life of Tumor Treating Fields (TTFields) therapy in glioblastoma. This unique noninvasive treatment uses a portable device to deliver electric fields to the tumor using arrays that are placed on the skin and kill cancer cells in several ways.

**Implications for Practice**

This article comprehensively reviews the mechanism, efficacy, safety, and quality of life of Tumor Treating Fields (TTFields) therapy in glioblastoma. This unique treatment uses a portable device to deliver electric fields to the tumor using arrays that are placed on the skin. TTFields specifically kill cancer cells in several ways, including enhancement of the body’s immune response against cancer cells. There is considerable evidence that TTFields therapy extends survival for patients with glioblastoma, while maintaining quality of life, and new clinical studies are expected to widen its use in the central nervous system and other solid tumors.

## Introduction

Glioblastoma (GBM) is the most common primary central nervous system (CNS) malignancy.^1^ In addition to surgery, radiotherapy (RT), and chemotherapy, the noninvasive, locoregionally applied, anticancer treatment modality of Tumor Treating Fields (TTFields) therapy (Optune Gio) is approved for newly diagnosed (nd) GBM (concomitant with maintenance temozolomide [TMZ] chemotherapy) and recurrent (r) GBM (as a monotherapy), in the United States (by the US Food and Drug Administration [FDA]), Canada, China, Israel, Japan, Australia, and several countries in Europe (the device is Conformité Européenne [CE]-marked by the European Union [EU]).^1-8^ In this paper, we describe studies showing that the survival of patients with ndGBM markedly improves with the use of TTFields therapy, alongside reviewing additional novel strategies with TTFields therapy that may further leverage its benefit for patients with GBM.

**Figure 1.**
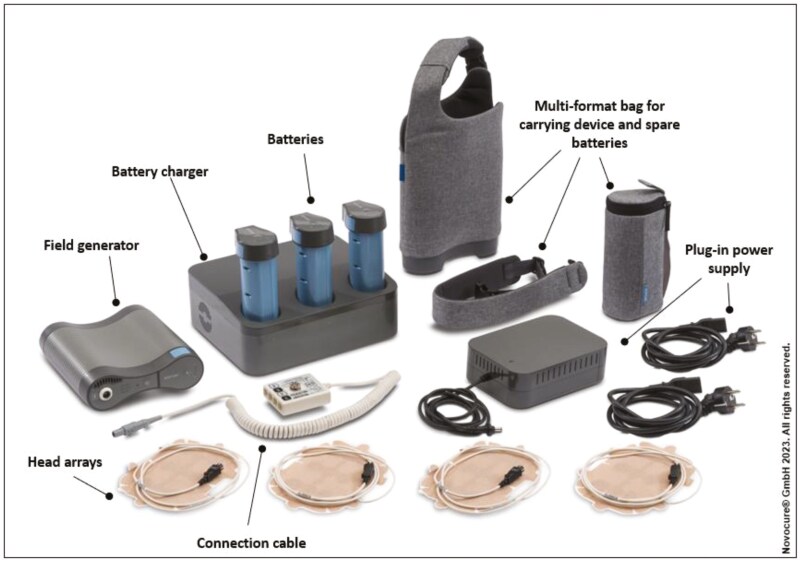
The second-generation Tumor Treating Fields therapy medical device system. The NovoTTF-200A (Optune Gio) system consists of a portable field-generating device (with a bag), arrays, batteries, battery charger, and plug-in power supply. This system is smaller and lighter than the originally introduced NovoTTF-100A device (1.2 vs 2.7 kg).^9^ Reused with permission from 2024 Novocure GmbH—all rights reserved.

TTFields therapy is based on the delivery of alternating electrical fields generated by a portable battery-powered device, that are delivered to the tumor by 4 (2 pairs) transducer arrays placed on the patient’s skin surrounding the tumor location (Figure 1). The array placement is determined based on individualized patient anatomy, with a corresponding layout defined by the treatment planning software. The portability of the device that generates TTFields allows for its use at home and during routine activities, thus incorporating it into the daily life of patients. Patients using TTFields therapy also benefitted from the introduction of a second-generation device that received a CE mark in 2015, and US FDA approval in 2016, and is smaller, lighter, with longer battery life, and uses less conspicuous, patient-centric tan arrays compared to the original device.^8,10^

### TTFields act via a multimodal mechanism of action

**Figure 2.**
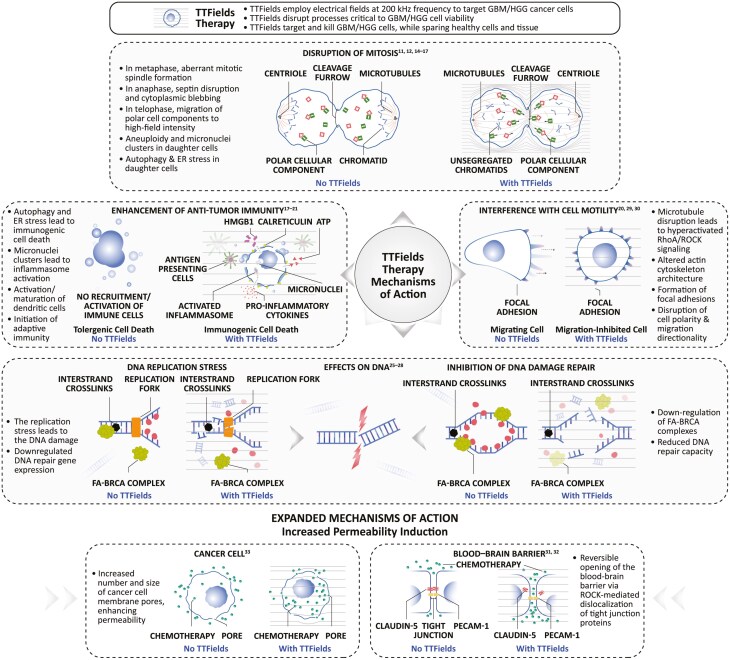
The anticancer effects of TTFields originate from a multimodal mechanism of action. TTFields multimodal mechanism of action impairs mitosis, the DNA damage response, and cell motility, and enhances antitumor immunity and cell and BBB permeability. Reused with permission from 2024 Novocure GmbH—all rights reserved. Abbreviations: ATP, adenosine triphosphate; BBB, blood–brain barrier; BRCA, breast cancer protein 1 or 2; DNA, deoxyribonucleic acid; ER, endoplasmic reticulum; FANC, Fanconi anemia complementation group; GBM, glioblastoma; HGG, high-grade glioma; HMGB1, high mobility group box 1 protein; PECAM-1, platelet endothelial cell adhesion molecule 1; Rho, Ras-homologous protein family; ROCK, Rho-associated protein kinase; TTFields, Tumor Treating Fields.

TTFields are electric fields that exert physical forces to disrupt critical cellular processes, ultimately leading to cancer cell death.^11,12^ TTFields therapy acts via a multimodal mechanism of action that includes effects on mitosis, autophagy, the DNA damage response, cell adhesion and motility, stimulation of antitumor immune responses, and increased cell and blood–brain barrier (BBB) permeability (Figure 2). As such, TTFields therapy is clinically versatile in terms of its potential for use as a monotherapy, or as an adjuvant with numerous existing anticancer therapies.^13^

### Antimitotic effects

TTFields have been shown to disrupt mitosis via functional effects on polar cellular components (Figure 2).^11,14-17^ TTFields affect the polar tubulin subunit that forms microtubules, leading to a reduction in tubulin polymerization and, accordingly, impaired mitotic spindle assembly during metaphase.^12^ Additionally, TTFields interfere with septin localization to the midline of the mitotic spindle during anaphase, inducing aberrant exit from mitosis.^12,16^ The hourglass shape of the dividing cell in telophase creates a nonuniform electrical field with high alternating electric field intensity at the cleavage furrow, causing migration of polar cell components via dielectrophoresis.^12^ These effects lead to apoptosis and/or formation of aneuploid daughter cells that display increased endoplasmic reticulum (ER) stress and autophagy.^12,15,17^ Overall, these events result in reduced cancer cell replication, proliferation, and tumor growth.

### TTFields enhance antitumor immune responses via induction of immunogenic cell death

There is also an interplay between TTFields and the immune system as the aneuploid daughter cells resulting from aberrant mitosis develop ER subcellular stress, activate autophagy, and undergo immunogenic cell death.^17-20^ This, in turn, enhances the systemic antitumor immune response (Figure 2).^17,21^ To summarize the laboratory evidence, TTFields treatment induces the hallmarks of immunogenic cell death, including the release of high mobility group box 1 protein (HMGB1), calreticulin exposure on the cell surface, and autophagy-mediated ATP release (Figure 2).^17^ Several of these events have also been demonstrated in vivo when TTFields treatment was applied to rodent cancer models.^19,21^

Emerging evidence has also highlighted that TTFields can modulate the tumor microenvironment to promote antitumor immune responses.^22^ GBMs are generally regarded as “cold tumors” which express high levels of immune checkpoint proteins and are not highly immunogenic. However, recent evidence suggests that cold GBMs can be “heated up” to enable immune-mediated tumor control.^23^ The antitumor immune response triggered by TTFields includes the induction of dendritic cell maturation, enhanced cancer cell phagocytosis by dendritic cells, and the promotion of leukocyte recruitment.^17^ When TTFields were applied concomitant with immune checkpoint inhibitors (ICI) in a lung cancer mouse model, increased levels of cytotoxic T cells were reported in the tumor microenvironment, alongside elevated levels of splenic effector memory cytotoxic T cells.^21^ Importantly, T cells cultured in the presence of TTFields treatment retained cytotoxic properties comparable to controls.^24^

Additionally, TTFields have been shown to induce antitumor memory immunity (adaptive immunity) in in vitro and in vivo models via dual activation of STING and AIM2 inflammasomes alongside a type 1 interferon (T1IFN) response by a mechanism involving the formation of micronuclei. Similarly, it has been reported in patients with GBM that TTFields trigger activation of adaptive immunity via a T1IFN-based trajectory, and there is a gene panel signature of TTFields effect on T-cell activation and clonal expansion.^23^ Of further potential importance for GBM where macrophages are frequent, in vitro TTFields treatment skewed typical M2 (pro-tumoral phenotype) mouse macrophages toward an M1 (proinflammatory) phenotype, increasing the release of inflammatory meditators.^22^

### DNA damage response

As electrostatic forces underlie multiple physiological and pathological processes, TTFields’ effects in cancer extend to several other cellular processes. This includes that TTFields treatment inhibits the DNA damage response (Figure 2). The expression of DNA repair genes is downregulated by TTFields, including genes associated with the FA-BRCA damage response.^25-28^ As such, in vitro exposure to TTFields treatment leads to an increase in DNA double-strand breaks. The additional appearance of chromatid-type aberrations,^25^ as well as shorter newly replicated DNA and evidence of R-loop formation^26^ are all indicative of atypical DNA replication.^26^ Ultimately, cells exposed to TTFields adopted a vulnerable phenotype whereby replication stress is enhanced, and DNA repair capacity reduced, rendering cells more susceptible to DNA-damaging agents or agents that interfere with DNA repair.

### Impaired cell motility and migration

TTFields treatment also interferes with cancer cell motility via the regulation of microtubules and actin dynamics.^20,29^ TTFields-induced changes in microtubule organization activate the RhoA/ROCK signaling pathway, which results in actin bundling and downstream formation of focal adhesions. The altered actin architecture and the decreased number of microtubules disrupt cell polarity, thus impacting the direction and the velocity of cell migration (Figure 2).^29^ Together with the activation of the immune system, these mechanisms may underlie the antimetastatic effects of TTFields observed in animal models.^29,30^

### Increase in cell and tissue permeability

TTFields treatment has been associated with several other cellular changes. One is that its application transiently and reversibly increased the permeability of the BBB in vitro and in vivo.^31,32^ This occurs via a molecular pathway originating from microtubule disruption that includes Rho kinase-mediated phosphorylation of the claudin-5 component of tight junctions, leading to dislocalization of tight junction proteins (claudin-5 and ZO-1) into the cytoplasm (Figure 2). By extension, it is theorized that molecules which poorly traverse the highly selective, semipermeable BBB under normal conditions may subsequently do so more effectively. This could potentially increase local concentrations of some therapies in the CNS.^31,32^ There are also in vitro data suggesting that TTFields treatment can induce pore formation in the plasma membrane of cancer cells, increasing permeability to small molecules (relevant for many anticancer treatments), whilst noncancerous cells remain intact.^33^

### Preclinical data on TTFields use in GBM models

Studies examining different frequencies of TTFields have determined that they have the greatest influence on GBM cells when used at 200 kHz.^11,14^ In addition, the anticancer effects of TTFields (at 200 kHz) in cultured GBM cells (evaluated using markers of cell viability, apoptosis, DNA damage, and/or mitotic abnormalities), were consistently enhanced when TTFields were applied concomitantly with other widely used GBM therapies, such as TMZ and RT.^20,34,35^ In vitro data also showed enhanced efficacy when including TTFields treatment with TMZ plus lomustine (CCNU); a combination chemotherapy regimen that has shown promise for ndGBM featuring *O*6-methylguanine-DNA methyltransferase (*MGMT*) promoter methylation^28^ and is being tested in ongoing trials (NCT05095376).

New insights into the use and impact of TTFields treatment on GBM tumors are being gained from next-generation in vitro culturing systems. One study examined the effects of TTFields applied to different three-dimensional ex vivo culture systems derived from patients with GBM: (1) GBM cells seeded onto mouse organotypic hippocampal slices to form microtumors, (2) GBM organoids, and (3) an organotypic GBM slice culture generated from fresh intra-operative material.^36^ The microtumors formed in these systems showed higher sensitivity to TTFields treatment than monolayer GBM cultures, in terms of tumor growth, viability, and the percentage of proliferating cells. The results also suggested that inter-patient differences in response were preserved in the models, especially the patient-derived organoids and tumor slice cultures.^36^

The magnitude of the anticancer effects of TTFields is dependent on the frequency, field intensity, time, and direction of TTFields delivery.^14,15^ Preclinical data have demonstrated that an effective field intensity is considered to be at least 1 V/cm in the region of interest, and data from computational modeling simulations have shown that these intensities are achievable in GBM with a variety of tumor sizes, shapes, and locations^11,14,37^ Importantly, there is no evidence of thermal damage to the skin associated with meaningful field intensities of 1 V/cm or more.^38,39^

## Clinical data on the use of TTFields therapy

### Efficacy findings in ndGBM

The European Organisation for Research and Treatment of Cancer (EORTC) protocol of maximally safe surgical resection of the tumor, followed by concurrent RT and chemotherapy (with TMZ), and then 6 months of adjuvant maintenance TMZ chemotherapy has been the backbone of ndGBM treatment.^4,40^ Since the Stupp protocol was published in 2005, only 2 pivotal/phase III studies enrolling patients of any age have reported significantly improved overall survival (OS) for ndGBM; one of which was the EF-14 study of TTFields therapy.^41,42^ TTFields therapy is currently approved in several countries, including the United States,^7^ for use with maintenance TMZ, and has a CE mark in the EU (grade 4 glioma) for use with either maintenance TMZ or a combination of TMZ plus lomustine therapy.^8,28^

The global randomized, pivotal (phase III) EF-14 clinical study (NCT00916409; Table 1) enrolled patients with newly diagnosed and histologically confirmed supratentorial GBM (World Health Organization [WHO] grade 4 astrocytoma), and demonstrated significantly improved survival when TTFields therapy was given concomitant with maintenance TMZ versus TMZ alone. The primary endpoint of progression-free survival (PFS) showed a median of 6.7 versus 4.0 months, and a hazard ratio (HR) of 0.63 (95% CI 0.52-0.76; *P* < .001), and the secondary endpoint of median OS was 20.9 months versus 16.0 months with a HR of 0.63 (95% CI 0.53-0.76; *P* < .001).^42^ The study also showed this was a long-term benefit: the 2-year survival rate was 43% (TTFields/TMZ) versus 31% (TMZ alone), and 5-year survival was more than doubled by including TTFields therapy (13% vs 5%).^42^ It should be noted that since the EF-14 study enrolled patients before the 2016 WHO reclassification of grade 4 gliomas to isocitrate dehydrogenase (IDH)-mutant and wild type, the patient population included both subsets of tumors (IDH-mutant: 7% [TTFields/TMZ] vs 5% [TMZ alone]; IDH wild type: 92% [TTFields/TMZ vs 95% [TMZ alone]).^42^ TTFields therapy is not mutationally driven and therefore, an OS benefit was seen regardless of *MGMT* promoter methylation status, with a more pronounced benefit observed in patients with *MGMT* promoter methylation (methylated *MGMT* [TTFields/TMZ]: median OS: 31.6 months, interquartile range: 21.1-48.5; unmethylated *MGMT* [TTFields/TMZ]: median OS: 16.9 months, interquartile range: 9.7-28.2).^42^ A subgroup analysis showed that the clinical benefit of concomitant TTFields therapy was present in older patients (≥65 years of age) who typically have poorer prognosis and are less able to tolerate traditional systemic treatments (Table 1). The EF-14 clinical study enrolled patients inside and outside of the United States, and found a similar clinical benefit in both populations,^42^ as well as in a specific subgroup analysis of patients enrolled in Korea^45^ (Table 1).

**Table 1.** Efficacy results from clinical studies of TTFields therapy in adult patients with ndGBM.

Study name; registration Region (enrollment years)	Study type	Therapy	*N*	Median OS	OS HR (95% CI) *P* value	2-year OS rate	5-year OS rate	Median PFS	PFS6	Reference
EF-07 Czech Republic	Prospective pilot	TTFields + TMZ	10	>39 mo	—	—	—	155 weeks	—	^43^
EF-14; NCT00916409 Global (2009-2014)^a^	Pivotal (phase III) randomized controlled	TTFields + TMZ vs TMZ	466 229	20.9 mo 16.0 mo	0.63 (0.53-0.76) *P* < .001	43% 31%	13% 5%	6.7 mo 4.0 mo	56% 37%	^42^
Elderly patients (≥ 65 years)	Subgroup analysis		89 45	17.4 mo 13.7 mo	0.51 (0.33-0.77) *P* = .02	39% 27%	15% 0%	6.5 mo 3.9 mo	53% 26%	^44^
Korean patients	Subgroup analysis		24 15	27.2 mo 15.2 mo	0.27 (0.10-0.75) *P* = .01	60% 30%	—	6.2 mo 4.2 mo	—	^45^
Usage (> 90% use vs ≤90% use)	Subgroup analysis		43 229	24.9 mo 16.0 mo	0.52 (0.35-0.79) *P* < .001	55% 31%	29% 5%	8.2 mo 4.0 mo	—	^46^
United States (2014-2017)	Retrospective	TTFields + TMZ vs TMZ	37 67	—	0.93 (0.58-1.47) *P* = .74	—	—	—	—	^47^
United States (2014-2017)	Retrospective	TTFields + CT^b^ vs CT	55 57	25.5 mo 18.8 mo	0.54 (0.31-0.94) *P* = .03	—	—	15.8 mo 6.9 mo	—	^48^
United Kingdom (2017-2019)	Prospective observational pilot^c^	TTFields + CT vs CT	9 9	14.9 mo 11.6 mo	Log-rank test; *P* = .39	—	—	5.5 mo 3.3 mo	—	^49^
EF-29 Japan (2016-2020)	Retrospective	TTFields + CT	40	NR	—	54%	—	15.8 mo	78%	^50^
China (2018-2021)	Retrospective	TTFields + TMZ vs TMZ	63 204	21.8 mo 15 mo^d^	0.43 (0.38-0.67) *P* < .001	—	—	16 mo 11 mo	-	^51^
Austria (2016-2021)	Retrospective	TTFields + TMZ	48^d^	22.6 mo	—	—	—	—	-	^52^
United States (2015-2021)	Retrospective	TTFields + TMZ vs TMZ	59 32	20.7 mo 15 mo	*P* = .04	33%	—	—	-	^53^
Czech Republic (2004-2021)	Retrospective^f^	TTFields + TMZ vs TMZ	55 54	31.7 mo 24.8 mo	0.61 *P* = .03	61% 53%	24% 12%	19.8 mo 12.5 mo	-	^54^
China (2013-2021)	Retrospective	TTFields + TMZ vs TMZ	13 39	24.8 mo 18.6 mo	*P* = .368	—	—	15.3 mo 10.6 mo		^55^
Global (2004-2020)^g^	Meta-analysis	TTFields + CT	512	21.7 mo	—	45%	—	7.2 mo^h^	56%	^56^
Germany (2012-2020)	Retrospective	TTFields + lomustine/TMZ vs lomustine/TMZ^i^	22 48	NR 26.7 mo	2.55 (1.25-5.20) *P* = .01	—	—	21.5 mo 11.2 mo	—	^57^
2-THE-TOP; NCT03405792 United States (2018^f^-2022)	Prospective pilot (phase II)	TTFields + pembrolizumab/TMZ vs TTFields + TMZ^j^	26 26	24.8mo 14.7mo	0.39 (0.19-0.78) *P* = .039	52% 12%	—	12.0 mo 5.8 mo	—	^58^
NCT03780569 Israel (Apr-Dec 2017)	Prospective pilot	TTFields + RT + TMZ	10	NR	—	—	—	8.9 mo	58%	^59^
SPARE; NCT03477110 United States (2018-2021)	Prospective pilot	TTFields + scalp preserving RT/TMZ	30	15.8 mo	—	—	—	9.3 mo	—	^60^
PriCoTTF Germany (2018-ongoing)	Prospective pilot/ (phase I/II)	TTFields + RT/TMZ	33	NR	—	—	—	—	—	^61^
Global (2020-2023)	Meta-analysis of real-world evidence	TTFields + CT vs CT	282 453	22.6 mo 17.4 mo	0.66 0.54-0.82 *P* < .001	47% 32%				^62^

The table shows studies that report outcomes from at least 10 patients. CT refers to treatment regimens where the study publication did not specify the type of chemotherapy or allow more than one CT regimen.

^a^In EF-14, PFS was the primary endpoint and OS was a secondary endpoint.

^b^The majority of patients received TMZ.

^c^Enrolled patients with poor prognostic markers who lacked promising treatment options.

^d^The control arm reports patients treated between 2016 and 2017.

^e^One patient had rGBM.

^f^Eleven patients were part of EF-07 and 8 were part of EF-14.

^g^Time period estimated from studies included in the analysis.

^h^Pooled PFS was calculated from 522 patients.

^i^Control arm includes patients who received TTFields for 0-8 weeks.

^j^Case controls from the EF-14 study.

Abbreviations: CT, chemotherapy; HR, hazard ratio; mo, months; ndGBM; newly diagnosed glioblastoma; NR, not reached; OS, overall survival; PFS, progression-free survival; PFS6, 6-month PFS rate; RT, radiotherapy; TMZ, temozolomide; TTFields, Tumor Treating Fields.

Real-world clinical studies have consistently supported the survival benefit detected in EF-14 (Table 1), including a retrospective study in the United States that reported longer OS with TTFields/TMZ versus TMZ alone (median OS 20.7 vs 15 months),^53^ and another for TTFields therapy with chemotherapy versus chemotherapy alone (median OS 25.5 vs 18.8 months)^48^ (Table 1). A retrospective analysis of 109 patients in the Czech Republic, including some who had participated in either an early pilot study (EF-07) or EF-14, reported median OS of 31.7 versus 24.8 months for TTFields/TMZ versus TMZ alone.^54^ Also, 2 retrospective analyses from Chinese institutes reported median OS of 21.8 versus 15.0 months (>200 patients),^51^ and 24.8 versus 18.6 months (~50 patients),^55^ for TTFields/TMZ versus TMZ alone.

Two meta-analyses of published clinical study data have also examined the survival benefit of adding TTFields therapy to maintenance chemotherapy. The first (published in 2021) calculated pooled OS from 4 studies (512 patients) and identified a median OS of 21.7 months for patients with ndGBM who received TTFields therapy with chemotherapy.^56^ A second more recent meta-analysis pooled OS data from 7 studies (1430 patients). Patients had received TTFields therapy with maintenance chemotherapy or maintenance chemotherapy alone.^62^ Pooled OS was significantly longer with TTFields therapy (HR 0.63; *P* < .001). Importantly the significant survival benefit was also detected when the analysis only considered data collected in real-world studies (median OS 22.6 vs 17.4 months; HR 0.66; *P* < .001), confirming that the efficacy identified by EF-14 translates to the routine clinic setting^62^ (Table 1).

Although the Stupp protocol for ndGBM is well established, modifications and new treatments are needed as the disease invariably recurs, and often within a year of initial treatment (Table 1). Several studies have investigated how to further leverage the benefit of using TTFields therapy in ndGBM. Particularly notable is a study of patients with ndGBM and positive *MGMT* promoter methylation where TTFields therapy for at least 8 weeks concomitant with combination TMZ and lomustine chemotherapy extended OS and PFS compared to patients who received TTFields therapy for less than 8 weeks or had no TTFields therapy. After a median follow-up of 25 months, the median OS was not reached for patients receiving 8 weeks or more of TTFields therapy, while it was 26.7 months for patients with less than 8 weeks (or no) TTFields therapy, and median PFS was 21.5 versus 11.2 months^57^ (Table 1). Additionally, a pilot (phase II) study (2-THE-TOP; NCT03405792) of TTFields therapy concomitant with pembrolizumab (an anti-PD-1 antibody) and TMZ combination therapy showed promising efficacy, with a median OS of 24.8 months and 2-year survival of 52%^58,63^ (Table 1). A randomized, controlled, pivotal (phase III) study examining TTFields therapy concurrent with pembrolizumab and TMZ in ndGBM (EF-41/KEYNOTE D-58) is planned to support these positive findings.^64^

There is also ongoing work examining the benefit of initiating TTFields therapy with the chemoradiation phase of the Stupp protocol. There are preclinical data showing that TTFields render cells more susceptible to DNA-damaging agents, including radiation therapy.^25,28^ The first pilot clinical study in patients (NCT03780569) reported a median PFS of 8.9 months with the addition of TTFields therapy^59^ (Table 1), while median OS was not yet reached in the pilot PriCoTTF study (which administered TTFields therapy prior to and concomitant with RT) after a median 8.9 months of treatment duration^61^ (Table 1). A randomized, controlled pivotal (phase III) study (TRIDENT; EF-32; NCT04471844) comparing the efficacy and safety of TTFields therapy concomitant with chemoradiation and maintenance TMZ, versus chemoradiation alone, followed by TTFields therapy and maintenance TMZ in all patients,^65^ has completed its target enrollment of 950 patients.^66^ This approach of initiating TTFields therapy earlier in the ndGBM treatment regimen may particularly benefit patients who would otherwise progress quickly on TMZ after radiation therapy, potentially including those with *MGMT*-unmethylated disease.^67^ Other approaches are also being studied, including TTFields therapy added to particle beam RT,^68^ and TTFields therapy provided using a modified scalp preserving regimen that also delivers RT through the TTFields arrays. For the latter, results from a 30-patient study showed a median PFS of 9.3 months, median OS of 15.8 months, and 1-year survival of 66% (NCT03477110).^60^

### Efficacy findings in rGBM

GBM invariably progresses or recurs. There is no standard of care at recurrence and systemic therapy options are primarily TMZ rechallenge, nitrosoureas, or anti-angiogenic (bevacizumab) therapy, but all lack efficacy and are associated with significant toxicities.^4,69,70^ TTFields therapy as monotherapy is approved for use in rGBM in many countries based on data from the randomized, global, pivotal (phase III) EF-11 study (NCT00379470) that showed TTFields therapy had comparable OS to physician’s choice of systemic therapy (a number of different agents), with a median OS of 6.6 versus 6.0 months^71^ (Table 2). Importantly, although survival was comparable between the 2 arms, TTFields therapy had a more favorable safety profile and better quality of life than found in the chemotherapy arm.^71^

**Table 2:** Efficacy results from clinical studies of TTFields therapy in adult patients with rGBM.

Study name; registration Region (enrollment years)	Study type	Therapy	*N*	Median OS	OS HR (95% CI) *P* value	1-year OS rate	2-year OS rate	Median PFS	PFS6	Reference
EF-07 Czech Republic	Prospective pilot	TTFields	10	62 weeks	—	68%	—	26 weeks^a^	50%	^14^
EF-11; NCT00379470 Global (2006-2009)	Pivotal (phase III) randomized controlled	TTFields vs CT^b^	120 117	6.6 mo 6.0 mo	0.86 (0.66-1.12) *P* = .27	20% 20%	8% 5%	2.2 mo 2.1 mo	21.4% 15.1%	^71^
PRiDe United States (2011-2013)	Retrospective registry	TTFields	457	9.6 mo	—	44%	30%	—	—	^72^
United States (2011-2013)	Retrospective	TTFields + BEV	34	4.1 mo	—	—	—	2.8 mo	—	^73^
United States (2013-2014)	Retrospective	TTFields + stereotactic radiosurgery vs TTFields	28 12	12 mo 4 mo	—	—	—	—	—	^74^
EF-14; NCT00916409 Global (2009-2014)	Post hoc analysis^c^ Pivotal (phase III) randomized controlled	TTFields + CT CT	144 60	11.8 mo vs 9.2 mo	0.70 (0.48-1.00) *P* = .049	—	—	—	—	^75^
United States (2013-2017)	Prospective Pilot (phase II)	TTFields + BEV	23	10.5 mo	—	46%	—	4.1 mo	33%	^76^
EF-19 United States (2016-2017)	Prospective registry	TTFields	192	7.4 mo	—	33%	—	3.3 mo^d^	—	^77^
United States (2011-2018)	Retrospective	TTFields + TBI	18	18.9 mo				10.7 mo		^78^
United States (2011-2018)	Retrospective	TTFields + BBC	30	11.8 mo				4.7 mo		^78^
United States (2010-2019)	Retrospective	TTFields + CT^e^ vs CT^e^	29 120	13.9 mo^f^ 10.9 mo^f^	*P* = .068					^79^
China (2013-2021)	Retrospective	TTFields vs CT	13 28	10.6 mo 13.3 mo	*P* = .655	39% 62%	—	8.4 mo 8.0 mo		^55^
Global (2004-2020)^g^	Meta-analysis	TTFields + CT	984	10.3 mo		43.7%	21.3%	5.7 mo^h^	48%	^56^
NCT02893137 Denmark (2016-2019)	Prospective pilot (phase I)	TTFields + skull remodeling surgery/CT^i^	15	15.5 mo	—	55%	—	4.6 mo	36%	^80^

The table shows studies that report outcomes from at least 10 patients. CT refers to treatment regimens where the study publication did not specify the type of chemotherapy or allowed more than one CT regimen.

^a^Time to progression (not PFS).

^b^Most patients received single agent or a combination chemotherapy regimen containing BEV (31%), or irinotecan (31%), followed by nitrosoureas (25%), carboplatin (13%), TMZ (11%), or various other agents (5%).

^c^Patients enrolled in EF-14 for ndGBM after disease progressed on either TTFields + TMZ or TMZ alone.

^d^Time to treatment failure (not PFS).

^e^CT was TMZ rechallenge, BEV, and/or irinotecan.

^f^Defined as post-progression survival.

^g^Time period estimated from studies included in the analysis.

^h^Pooled PFS was calculated from 201 patients.

^i^Best choice chemotherapy was either BEV alone, BEV + lomustine, BEV + irinotecan, or TMZ rechallenge.

Abbreviations: BEV, bevacizumab; CCNU, 1-(2-chloroethyl)-3-cyclohexyl-1-nitrosourea; CT, chemotherapy; HR, hazard ratio; mo, months; NR, not reached; OS, overall survival; PFS, progression-free survival; PFS6, 6-month PFS rate; rGBM; recurrent glioblastoma; RT, radiotherapy; TBI, temozolomide + bevacizumab + irinotecan; TMZ, temozolomide; TTFields, Tumor Treating Fields.

Real-world data largely support TTFields therapy use in rGBM, including 2 large registry studies (PRiDe and EF-19) that showed longer OS with TTFields therapy in clinical practice than in either arm of the EF-11 study (9.6 and 7.4 months), although noting that PRiDe did not capture information on concomitant therapy use^72,77^ (Table 2). New and optimized therapies are being evaluated for rGBM, including a study underway exploring TTFields therapy concomitant with stereotactic radiosurgery (TaRRGET; NCT04671459),^81^ that will hopefully provide additional benefit for patients.

There is also evidence to support that the current rGBM indication of TTFields therapy as monotherapy should be expanded to include TTFields therapy given concomitant with chemotherapy. This includes post hoc analysis of patients in the EF-14 study after disease progression on study chemotherapy (TMZ), which found significantly longer OS in patients who received TTFields therapy with second-line chemotherapy compared to patients who received chemotherapy alone (median OS 11.8 months vs 9.2 months).^75^ It should be noted, however, that although the findings of the EF-14 post hoc analysis can be broadly applied to rGBM, the population studied was different to that in EF-11. Patients in the EF-14 post hoc analysis had experienced disease progression after initiating TTFields therapy, but continued TTFields therapy during first-line maintenance TMZ.^75^ Conversely, the population studied in EF-11 had not been treated with TTFields therapy with initial adjuvant TMZ.^71^ In addition, a real-world evidence study has shown a median post-progression survival of 13.9 versus 10.9 months for patients with rGBM receiving TTFields therapy and chemotherapy versus patients receiving only chemotherapy^79^ (Table 2).

### Several factors influence the efficacy of TTFields therapy

Unlike systemic therapies, there are no pharmacokinetic or pharmacodynamic considerations for the use of TTFields therapy, it is not impeded by the BBB and efficacy does not appear to require a specific mutational or biomarker status. Indeed, as a physical modality, TTFields therapy is an ideal adjuvant treatment. It also appears that since TTFields therapy is not mutationally driven, it has efficacy in a broad set of patients and does not strongly correlate with any specific patient or GBM tumor characteristic, with the exception of factors that would normally translate into better outcomes. Specifically, there appears to be a more pronounced benefit in patients <65 years of age (vs those ≥65 years of age), higher Karnofsky performance score (90-100 vs ≤80), and in patients with *MGMT* promoter methylation (vs those without *MGMT* promoter methylation).^42^ Although relationships with tumor biomarker and genetic status have been suggested, results have been highly inconsistent and should be interpreted cautiously given the heterogeneous and relatively small populations examined.^48,55,79,82^

There is, however, a clear correlation between the efficacy of TTFields therapy and the duration and dose of fields delivered to the tumor. In the EF-14 clinical study of ndGBM, ≥50% average monthly use was required for extended OS (HR 0.67 [95% CI 0.45-0.99]), and patients with >90% had a 5-year survival of almost 30%, a landmark result in this patient population^46^ (Table 1). In rGBM (the EF-11 study), a longer median OS was associated with patients using TTFields therapy for an average of ≥75% of each day (≥18 hours/day) versus <75% of each day (7.7 vs 4.5 months; log-rank *P* = .04).^83^ Meta-analysis of pooled data from randomized and real-world studies has also confirmed the relationship between TTFields therapy use and survival. In ndGBM, mean use of ≥ 75% was associated with prolonged survival (HR 0.60; *P* < .001,^62^ and in rGBM 262 patients with ≥75% TTFields therapy use had pooled median OS of 10.3 months, while 286 patients with <75% use had pooled median OS of 5.7 months.^56^

Most patients with GBM in clinical studies of TTFields therapy have achieved the minimum recommended usage that correlates with improved outcomes (77% and 57% of patients in EF-11 and EF-14 had ≥75% usage time, respectively).^42,83^ Real-world studies suggest similar rates are achievable, including in Japan where 52.5% of 1066 patients treated between 2016 and 2019 achieved 75% usage time.^84^ As discussed below, new practical guidelines and prevention and management approaches and strategies to minimize localized skin irritation associated with TTFields therapy arrays or hydrogel are expected to facilitate increased usage time moving forward.

Analogous to the calculation of RT dosing, the energy imparted by TTFields therapy to a tumor can be described in terms of power density and field intensity. A post hoc analysis from the EF-14 clinical study (ndGBM) calculated these for each individual patient using realistic head models derived from magnetic resonance imaging scans, integrated with the specific array layout, average usage time, and electrical current intensity delivered to the patient.^85^ Survival was significantly longer in patients who received an average local minimum power density in the clinical target volume of ≥1.15 mW/cm^3^ versus those receiving <1.15 mW/cm^3^ (median OS 24.9 vs 21.5 months; HR 0.69; *P* = .01).^85^ Tumor progression also occurred at greater distances from the primary tumor in patients receiving TTFields therapy, and brain areas that showed tumor regression had received a significantly higher average field intensity than areas where tumor progression occurred.^86^ Approaches that optimize TTFields delivery to the tumor are currently being evaluated, including a prospective pilot (phase I) study examining the feasibility of TTFields therapy concomitant with targeted personalized skull remodeling surgery designed to accumulate the TTFields dose intensity focally in the tumor.^80^ Pilot data showed the approach to be safe and nontoxic, and demonstrated a median OS of 15.5 months in patients with rGBM (Table 2). A larger study of 70 patients is in progress (OptimalTTF-2; NCT04223999).^87^

### The safety profile of TTFields therapy is consistently limited to mild-to-moderate skin reactions

As described below, clinical studies in GBM have consistently shown that adverse events (AEs) related to TTFields therapy were mostly limited to manageable and reversible mild-to-moderate skin irritation beneath the arrays. There is no evidence for added systemic toxicities. This includes that the EF-11 clinical study (rGBM) found more high-grade (Common Terminology Criteria for Adverse Events grade ≥3) gastrointestinal (3% vs 1%), hematologic (4% vs 0%), and infectious AEs (1% vs 0%) in patients receiving chemotherapy versus those receiving TTFields therapy^71^; and in the EF-14 study (ndGBM), the frequency of systemic AEs was comparable between the TTFields/TMZ versus TMZ alone groups (48% vs 44%),^42^ highlighting that systemic AEs were likely due to the chemotherapy. This observation is supported by a pooled analysis of 12 studies and 11 558 patients who had received TTFields therapy.^56^

**Figure 3.**
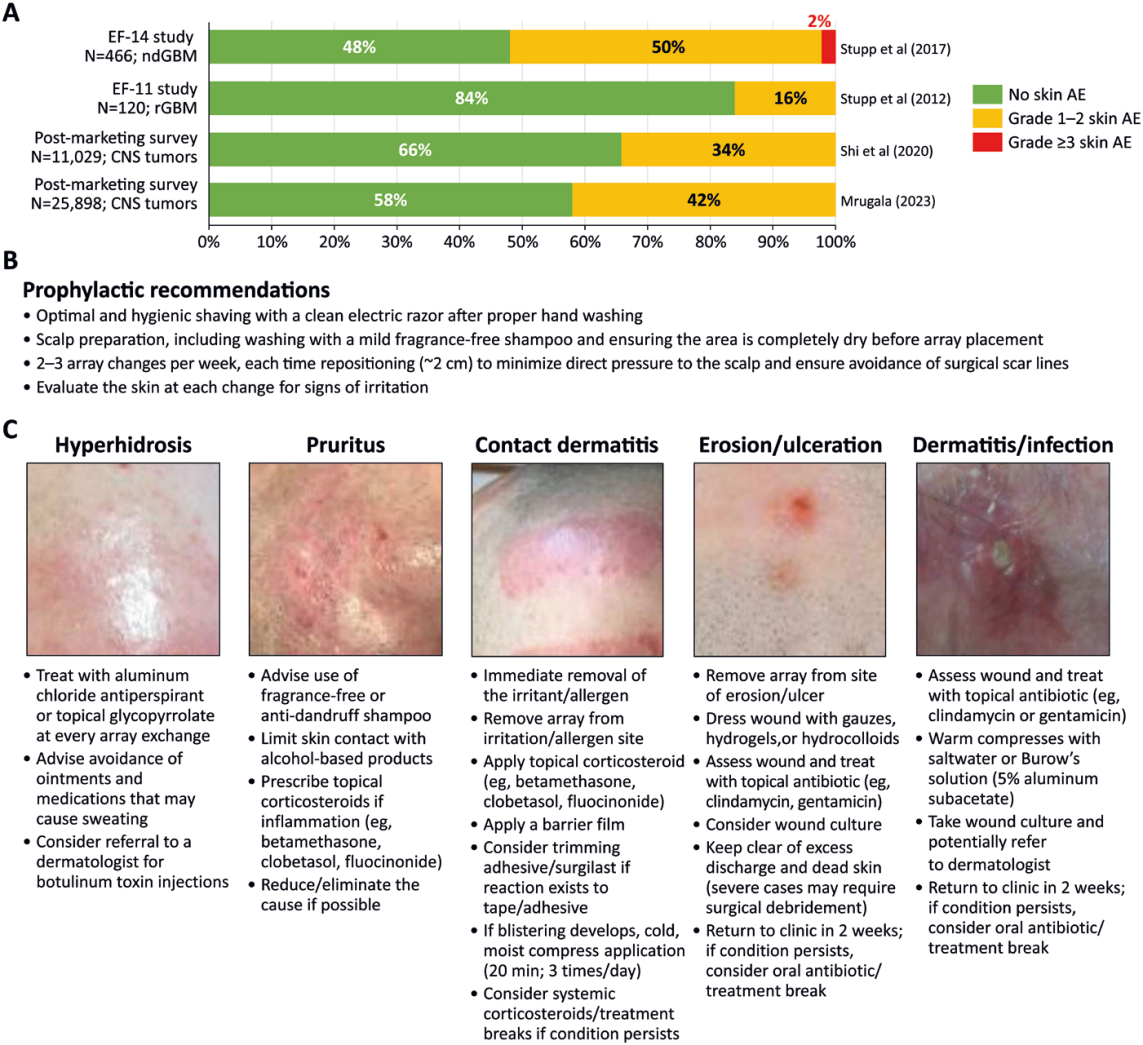
Summary of safety findings in Tumor Treating Fields studies. A) Skin safety findings in key studies. B) Recommendations to avoid skin AEs. C) Prevention and management strategies for skin AEs on the scalp, republished from Lacouture et al (2020)^88^ Copyright 2020 Lacouture, Anadkat, Ballo, Iwamoto, Jeyapalan, La Rocca, Schwartz, Serventi and Glas. Abbreviations: AE, adverse event; ndGBM, newly diagnosed glioblastoma; rGBM, recurrent glioblastoma.

Dermatological AEs associated with TTFields therapy occur beneath the arrays and can be attributed to skin contact with adhesive or hydrogel on the arrays.^88^ The Lacouture et al^88^ publication provides a good point of reference for identifying and managing dermatological AEs associated with TTFields therapy in patients with glioblastoma. Skin events are the most common AE associated with TTFields therapy (Figure 3A), and occurred in 16% of patients in the EF-11 study and 52% of patients in the EF-14 study receiving TTFields therapy; notably, there were no grade ≥3 dermatological AEs in the EF-11 study, and they occurred in only 2% of patients in the EF-14 study.^42,71^ Data from real-world practice show a similar safety profile, although it should be noted that real-world results are not subjected to the stringent collection protocols as large clinical studies and may therefore be less accurate. In the PRiDe registry (457 patients with rGBM treated in the United States 2011-2013), skin AEs were reported by 24% of patients and there were no new safety signals to those reported in the EF-11 study.^72^ The most recent analyses of data from global postmarketing surveillance datasets (one of >10 000 patients and one of >25 000 patients) reported skin AEs in 34% and 43% of patients receiving TTFields therapy for CNS tumors, no new safety signals, and that the frequency of AEs was consistent across diagnoses and age groups (Figure 3A).^89,90^ Of note, subgroup analyses of postmarketing surveillance data have shown a similar incidence of skin AEs in patients ≥70 years of age (*n* = 4071) with CNS malignancies receiving TTFields therapy compared with the overall population (*n* = 25 898; 45% vs 43%, respectively).^91^

Dermatological AEs associated with TTFields therapy include contact dermatitis, hyperhidrosis, xerosis or pruritus, and more rarely, skin erosions/ulcers and infections.^88^ Although normally mild-to-moderate, it is important to minimize their likelihood and to promptly manage those that occur, to avoid discontinuation and allow patients to achieve device usage associated with higher efficacy. New guidance was recently published that emphasizes the importance of careful application, removal, and repositioning of the arrays, together with regular examination of the skin on which they are placed (Figure 3B).^88^ Topical agents can be used to treat and manage most skin irritations, while patients with grade 2-3 skin AEs should be referred to a dermatologist (Figure 3C).^88^ These procedures are particularly important if patients have risk factors for TTFields therapy-associated AEs, such as a prior craniotomy, injury from radiation therapy, concomitant treatment with TMZ, steroids, bevacizumab therapy, or even preexisting skin conditions and persistent alopecia. An ongoing open-label study is examining prophylactic therapies for skin AEs with TTFields therapy in GBM (PROTECT; NCT04469075),^92^ with results expected to identify preventive measures that further reduce their frequency and severity.

Focused analyses in high-risk populations suggest that the good safety profile of TTFields therapy extends to a broad set of patients. For instance, in patients with GBM who required a ventriculoperitoneal (VP) shunt, there was no apparent difference in the frequency of localized skin AEs compared to the overall population (43% of 156 patients with a VP shunt), and no shunt malfunctions were considered related to TTFields therapy.^93^ Post hoc analysis of elderly patients in the EF-14 study also showed a comparable rate of grade ≥3 systemic AEs with TTFields/TMZ versus TMZ alone (46% vs 40%), and the only TTFields therapy-related AEs were reversible scalp skin reactions (51% patients reported grade 1-2, and 2% grade 3).^44^ The 2 large surveillance surveys also found similar frequencies of AEs in the elderly and overall patient population^89^; Shi et al^89^ reported at least one AE in 63% of adults 18-64 years of age (*n* = 8090) versus 66% of elderly patients (≥ 65 years of age; *n* = 2887), with skin irritation in 34% versus 36%, respectively. The datasets also had AEs reported by a small number of patients in pediatric care who received TTFields therapy off-label (approval is limited to adults per the populations enrolled in the EF-11 and EF-14 studies). Both found no difference in the frequency of AEs compared to adults; in the Shi et al^89^ study, 58% of pediatric cases (*n* = 52) reported at least one AE, of which 37% were skin AEs, and in the Mrugala et al^94^ study where 0.4% of 25 898 patients were classified as pediatric, the frequency of skin reactions was 39% versus 43% for all patients. Goldman et al^95^ further analyzed AEs in patients in pediatric care from the latter postmarketing surveillance safety dataset and found no difference in the rate of skin AEs between children <13 years of age (35%), and adolescents (13-17 years of age; 37%). A multicenter, single-arm pilot (phase I/II) study (NCT03033992) examining TTFields therapy in children with high-grade supratentorial glioma is underway, including examining TTFields therapy given concomitant with radiation therapy in cases with newly diagnosed diffuse intrinsic pontine glioma.^96^

To date, safety data collected by pilot studies examining TTFields therapy concomitant with other systemic and/or radiation therapies (Tables 1 and 2) continue to suggest safety is manageable. There were no high-grade skin AEs reported for TTFields therapy given concomitant with combination TMZ/lomustine (*n* = 22), and the rate of all high-grade AEs was comparable to that previously reported for chemotherapy alone.^57^ Patients with rGBM also tolerated TTFields therapy added to combination systemic therapy regimens (including triple combinations),^78^ although TTFields therapy with bevacizumab (anti-angiogenesis agent) should be used cautiously as it may impact wound breakdown and healing.^88^ The pilot study examining TTFields therapy concomitant with RT and TMZ in 10 patients with ndGBM reported skin AEs in 8 patients, but no other TTFields therapy-related AEs, and no increase in toxicities related to RT or TMZ^59^; this finding will be further examined in the pivotal TRIDENT study of TTFields therapy given with radiation therapy and TMZ.^65^

### TTFields therapy is associated with sustained quality-of-life findings

Studies exploring patient-reported outcomes (PROs) have largely confirmed the feasibility of using TTFields therapy in patients with GBM. There was no significant difference in health-related quality-of-life (QoL) scores between patients in EF-14 receiving TTFields/TMZ versus TMZ alone, with the exception of worsened itchy skin.^97^ Patients receiving TTFields/TMZ in EF-14 also had significantly better deterioration-free survival for physical and emotional functioning, global health, pain, and leg weakness, than patients receiving TMZ alone.^97^ The largest analysis of PROs to date was conducted on 1106 responses to a survey mailed to patients with GBM (ndGBM or rGBM) who received TTFields therapy during regular clinical care in the United States, Austria, Germany, or Switzerland.^98^ Regression analysis detected the expected correlations between worse QoL and disease progression and older age. The analysis also detected associations between a longer time using TTFields therapy and better mobility, self-care, participation in usual activities, and overall health scores.^98^ A 2-center study examining PROs from 30 patients who received TTFields therapy plus chemotherapy found that high use of TTFields therapy affected daily life at least 2-3 times per week, with device size, weight, and array replacement as the most frequently reported specific reasons. However, 70% of patients stated that they would recommend TTFields therapy to another patient, and 67% would use TTFields therapy again, based on their experiences.^99^ In another survey of patients in the United States (2018-2020), 97% of respondents identified the survival benefit as the key factor underlying their selection of TTFields therapy.^100^ Patients also indicated that knowledge of the extended survival would have an impact on their decision to initiate or remain on TTFields therapy. Additionally, there is evidence that health care practitioners perceive that patients are more challenged by TTFields therapy use than patients report for themselves, including for concerns about shaving the head (37% of health care practitioners vs 18% of patients), carrying the device (34% vs 18%), and visibility of the therapy (37% vs 24%).^101^

A number of real-world studies are underway that will give additional insight into the QoL of patients with GBM receiving TTFields therapy. Preliminary data from the prospective, noninterventional TIGER study (NCT03258021) found that, except for itchy skin, TTFields therapy did not impair health-related QoL in German patients with ndGBM.^102^ The TIGER PRO-Active study (NCT04717739) will report on the effect of TTFields therapy on QoL parameters including daily activity, sleep, and neurocognitive functioning, in German patients with GBM.^103^

### Guideline recommendations for the use of TTFields therapy

Like all therapies, the survival and QoL benefits demonstrated for TTFields therapy in GBM are frequently measured against the financial cost of the therapy. At the time of writing, and despite consistent results in clinical studies, clinical guidelines developed in different global regions have not yet achieved consistent recommendations for TTFields therapy in GBM. Guidelines for ndGBM include those from the European Association of Neuro-Oncology that note the positive efficacy and safety data for TTFields therapy given concomitantly with TMZ, but do not consider it as a primary standard of care; one reason being that the cost-effectiveness “remains highly controversial.”^104^ In contrast, the National Comprehensive Cancer Network (NCCN) recommends TTFields therapy with standard RT and concurrent/adjuvant TMZ as an NCCN category 1, preferred option for patients with GBM, supratentorial disease, and good performance status.^105^ “Category 1” is based upon high-level evidence (≥1 randomized phase III trials or high-quality, robust meta-analyses), and there is uniform NCCN consensus (≥85% support of the Panel) that the intervention is appropriate.^105^ Joint guidelines from the American Society of Clinical Oncology and Society of Neuro-Oncology (United States) state that TTFields may be added to maintenance TMZ, as a “weak” recommendation based on “moderate quality of evidence that benefits outweigh risks.”^106^ The joint Spanish Group of Investigation in Neuro-Oncology and Spanish Society of Medical Oncology guidelines recommend that clinicians should consider first-line treatment with TTFields therapy concomitant with TMZ, in patients with high-grade glioma without suspected pseudo/progression, following chemoradiation with TMZ.^107^ The same guidelines also highlight TTFields therapy as an option for patients with rGBM.^107^ The Commission Guidelines of the German Society for Neurology, used in Germany, Switzerland, and Austria, recommend TTFields therapy concomitant with TMZ maintenance therapy for patients with IDH wild type, WHO grade 4 glioblastoma, or diffuse hemispheric glioma, H3.3 G34-mutated, WHO grade 4.^108^ Furthermore, The Swedish National Care Program guidelines recommend that patients with supratentorially located grade 4 GBM without clinical signs of progression, who have completed chemoradiotherapy should be offered the addition of TTFields therapy for up to 2 years, or until disease progression, providing there are no contraindications.^109^ For rGBM, the European Association of Neuro-Oncology makes the recommendation that TTFields therapy should be discontinued if the disease progressed while patients were receiving TTFields therapy for ndGBM,^104^ despite the positive clinical data identified in the EF-14 study that again, support continuation of TTFields therapy beyond first progression (to second progression or 24 months), as a tolerated and effective treatment.^75^ American Society of Clinical Oncology-Society for Neuro-Oncology makes no recommendation for or against any therapeutic strategy for rGBM,^106^ while NCCN makes a category 2B recommendation to treat patients with recurrent or progressive GBM with TTFields therapy^105^ (“category 2B” being based upon lower-level evidence, and a consensus NCCN Panel vote of between 50% and 85% of members that the intervention is appropriate).^105^

### Health economic considerations of using TTFields therapy

The discordance between treatment guidelines may contribute to why not all patients who are eligible for TTFields therapy receive the treatment. A study published in 2020 reported that TTFields usage remained infrequent, with fewer than 20% of patients with GBM receiving the therapy at major academic medical centers in the United States and Germany,^110^ while in 2023 an analysis of electronic medical records from 726 patients with GBM in the United States reported that 30% had used TTFields therapy.^111,112^ There are also large differences between individual countries for reimbursement and provision of TTFields therapy. Some countries have health insurance systems that reimburse for TTFields therapy in ndGBM and rGBM, others for only ndGBM (including Switzerland,^113^ France,^114^ and Japan^89^), while some public health care systems have no provision of TTFields therapy for either ndGBM or rGBM.^115^

One possible reason for inconsistent guidelines and use of TTFields therapy is that few studies have examined the question of its cost-effectiveness, and results to date are highly inconsistent. The addition of TTFields to maintenance TMZ for ndGBM has been estimated to result in 0.3-0.6 and 1.3-1.8 incremental life years gained (LYG) in modeling studies for ndGBM based on the French and US health care systems, respectively.^116-119^ Thus, while an analysis from a US health care payer perspective demonstrated an incremental cost-effectiveness ratio (ICER) of ~$150 000/LYG,^116^ 2 analyses based on the French health insurance system calculated an ICER above €500 000/LYG^118,119^; in the latter analyses, there was zero probability of TTFields being cost-effective at a threshold of €100 000/LYG.^118,119^ Of note, the cost of TTFields treatment was a significant factor to which all models/analyses were sensitive^116,118,119^ and an 85% reduction in the cost of TTFields would be required to achieve an ICER under €100 000/LYG for ndGBM.^119^ To date, there has been even less analysis of the cost-effectiveness of TTFields therapy for rGBM. Ongoing studies on the efficacy of TTFields therapy concomitant with new rGBM therapies (Table 2) may help refocus perceptions about its cost-effectiveness.

### Future directions with TTFields therapy

The use of TTFields therapy continues to evolve. New guidance on minimizing skin irritation, continuing device improvements, and introduction of new arrays in Europe, are expected to help patients achieve the efficacy benefits of increased time on therapy.^8,88,120,121^ A new study is also underway testing approaches to minimize skin irritation.^92^ It is further likely that the use of TTFields therapy will expand when ongoing studies output results, including studies testing TTFields therapy administered with RT,^81^ immunotherapy,^64^ skull remodeling surgery,^87^ and in patients in pediatric care.^96,122^ In particular, results are eagerly awaited now that patient recruitment has been completed in the randomized controlled pivotal (phase III) TRIDENT study of TTFields therapy concomitant with chemoradiation in ndGBM.^65,66^

TTFields therapy is also being investigated for other types of tumors.^123,124^ In the CNS, the pivotal (phase III) METIS study (EF-25; NCT02831959) is assessing TTFields therapy following treatment with stereotactic radiosurgery (SRS) with best supportive care as compared to SRS and best supportive care alone in patients with brain metastases from non–small cell lung cancer.^125^ In addition, a pilot (phase I/II) study is examining TTFields therapy with the ICI, pembrolizumab, for newly diagnosed brain metastases from melanoma (NCT04129515).^126^ The randomized pivotal (phase III) LUNAR study (EF-24; NCT02973789) in patients with metastatic non–small cell lung cancer progressing on or after platinum-based therapy showed a clinically and statistically significant survival benefit for TTFields therapy added to an ICI or docetaxel versus ICI or docetaxel alone (median OS 13.2 vs 9.9 months).^127^ Interestingly, the survival benefit was particularly pronounced (~8 months) in the subgroup of patients receiving an ICI.^127^ These positive data further support the importance of the previously mentioned planned pivotal (phase III) study of TTFields therapy concurrent with pembrolizumab and TMZ in ndGBM (EF-41/KEYNOTE D-58).^64^ Additionally, on the basis of promising pilot data,^128-131^ pivotal (phase III) studies are currently underway in pancreatic (PANOVA-3; EF-27; NCT03377491) cancer,^132^ and are planned for hepatic and gastric cancer.

Ongoing computer modeling and simulation studies are investigating how to optimize TTFields therapy use in clinical practice, with 2 recent studies directly relevant to GBM. The first has developed a working framework for personalized segmentation-based treatment planning via the creation of a computational model of the head from segmented magnetic resonance imaging data, followed by the application of qualitative and quantitative tools to identify 2 layouts for each patient (to allow switching and thus minimize the risk of skin irritation) that achieve the optimal distribution of TTFields therapy.^133^ In addition, a separate study reported a comparison of segmentation-based treatment planning versus the currently used NovoTAL planning. This found that segmentation-based treatment planning delivered higher expected local minimum power density and field intensity to regions of interest than NovoTAL planning. Overall, the use of layouts designed by an optimized planning technique may help maximize outcomes with TTFields therapy in patients with GBM.^134^

## Conclusions

The survival benefit of adding TTFields therapy to TMZ in ndGBM was originally reported in the randomized, controlled pivotal (phase III) EF-14 study and has now been confirmed by studies of real-world clinical practice data, including in datasets collected at institutes in the United States, Asia, and Europe.^56,62^ In addition, reports from patients with rGBM support that survival results in the EF-11 study translate across to the real-world setting.^56,62^ Although some treatment guidelines may not have fully considered the evidence demonstrating improved survival for patients using TTFields therapy for ndGBM, NCCN recommends TTFields therapy with standard RT and concurrent/adjuvant TMZ as a NCCN category 1, preferred option for patients with GBM, supratentorial disease, and good performance status.^105^

TTFields therapy is not impeded by the BBB, has no pharmacokinetic considerations and, per evidence to date, its efficacy is not limited to tumors with specific molecular characteristics (eg, a genetic mutation). TTFields therapy has also shown no systemic toxicity to date, nor does it add to systemic toxicities when given concomitantly with a variety of systemic therapies. AEs related to the TTFields therapy device are limited to manageable, reversible, mild-to-moderate skin reactions (including in high-risk populations of the elderly, pediatric, and patients with VP shunts), and studies assessing health-related QoL found no impact except for itchy skin.

To summarize, TTFields therapy with a multimodal mechanism of action has demonstrated efficacy (OS and PFS), alongside sustained QoL and a consistently favorable safety profile, in patients with ndGBM and rGBM, and shows broad applicability across a range of solid tumors and patient populations.

## Data Availability

No new data were generated or analyzed in support of this research.
